# Sufficient stability using retrograde cannulated screws in a metacarpal fracture model: a biomechanical evaluation

**DOI:** 10.3389/fbioe.2025.1714404

**Published:** 2026-01-05

**Authors:** Maximilian Heilig, Julian Wagenhäuser, Henner Huflage, Philipp Heilig, Martin Cornelius Jordan, Rafael Gregor Jakubietz, Rainer Heribert Meffert, Stefanie Hoelscher-Doht

**Affiliations:** 1 Department of Trauma and Orthopaedic Surgery, BG Unfallklinik Frankfurt am Main, Frankfurt am Main, Germany; 2 Department of Trauma, Hand, Plastic and Reconstructive Surgery, University Hospital Würzburg, Würzburg, Germany; 3 Department of Diagnostic and Interventional Radiology, University Hospital Würzburg, Würzburg, Germany; 4 Center for Orthopedics, Trauma Surgery and Rehabilitation Medicine, Universitätsmedizin Greifswald, Greifswald, Germany

**Keywords:** metacarpal fracture, cannulated compression screw, plate, implant anchorage, cyclic loading

## Abstract

**Introduction:**

The treatment of metacarpal shaft fractures using cannulated compression screws has become a viable method of osteosynthesis in recent years. However, most biomechanical studies focus primarily on the ultimate failure load, comparing it to plating or K-wires. There is limited biomechanical data how cannulated compression screws perform under realistic cyclic loads encountered during postoperative physiotherapy in comparison to plating.

**Methods:**

Oblique shaft fractures were created in both porcine metatarsal and human metacarpal bones. Fractures were reduced and treated with either a partially or fully threaded cannulated compression screw or a 2.0 TriLock hand plate. Cyclic loading was then applied for 3,000 cycles, ranging from 10 to 80 N, followed by an ultimate load-to-failure test.

**Results:**

The displacement after cyclic loading was lower for specimens being treated with a 2.0 TriLock hand plate compared to those treated with an intramedullary compression screw. In the subsequent load-to-failure tests, specimens with a fully threaded intramedullary screw withstood higher forces than those with a partially threaded screw or a plate. Notably, no superiority of the plate was observed in terms of ultimate loading in both porcine and human specimens.

**Discussion:**

The results indicate that using a cannulated compression screw for the treatment of metacarpal oblique fractures provides good biomechanical stability under cyclic loading. Additionally, the use of a fully threaded screw appears to increase the ultimate failure load. This study adds to the biomechanical evidence supporting the use of cannulated compression screws in the treatment of metacarpal oblique fractures. Surgeons should prioritize achieving the best and longest cortical anchorage when selecting the implant to optimize the patient’s outcome.

## 1 Introduction

Metacarpal fractures are common in adult males aged 20 to 40, typically resulting from direct trauma, such as a fistfight, a fall, or a sports injury (Taha et al., 2024). While non-displaced fractures can be treated conservatively, displaced or misrotated fractures require surgical intervention. Four established methods are available for the operative treatment of these fractures: osteosynthesis using Kirschner wires, a dorsal plate, screws, or more recently, the use of an intramedullary, headless, cannulated compression screw (CCS) (Beck et al., 2019).

The first report of using an intramedullary, cannulated, headless screw for metacarpal fracture fixation was published by Boulton et al., in 2010, highlighting excellent functional outcomes (Boulton et al., 2010). The advantages discussed include the minimally-invasive surgery technique, which avoids extensive soft tissue dissection, the high primary and rotational stability due to fracture compression and the lower risk of implant-related complications. For the patient, it allows an earlier mobilization, leading to better functional outcomes and faster return to work. In addition, secondary implant removal is not mandatory for intramedullary screws, in contrast to plates that lie beneath the extensor tendons and may cause irritation. However, the required antegrade or retrograde approach through a healthy, cartilage-covered joint area has been controversially discussed. While only a small portion of the articular surface is affected, there are no long-term studies on the incidence of postoperative arthritis (Dohse et al., 2022; Ho et al., 2021; Straszewski et al., 2022). Clinical evidence supporting this approach continues to grow, with most studies reporting good outcomes and a low risk of implant or technique associated complications (Beck et al., 2019; Gehring et al., 2025; Supichyangur et al., 2022).

From a biomechanical perspective, intramedullary headless compression screws appear to be non-inferior to K-wires (Albanese et al., 2023; Graf et al., 2024). However, when compared to plating, the results are less conclusive. Some studies suggest a greater stability with plating (Galbraith et al., 2022; Labèr et al., 2020; Melamed et al., 2016), while others favor the intramedullary screw (Dyrna et al., 2021). A direct comparison can be misleading due to differences in technique, specimen types, and testing methods. Most published studies focus on the ultimate failure load, representing a worst-case scenario such as a fall or a direct impact during the initial postoperative phase. However, biomechanical data comparing the use of screws and plating in more realistic cyclic loading scenarios–such as postoperative physiotherapy involving functional finger movement without weight-bearing–remains limited (Jones et al., 2019).

We hypothesize that the use of cannulated compression screws is not inferior to plating under cyclic loading conditions, and thus, we aimed to test this hypothesis using an established fracture model.

## 2 Materials and methods

### 2.1 Specimen preparation

Specimen preparation followed a standardized protocol. To simulate a young and healthy cohort, freshly butchered pig feet were sourced from a local butcher and their metatarsal bones 2 and 5 were carefully dissected to remove all soft tissue. The biomechanical properties of these closely resemble those of human metacarpal bones and have been validated as an appropriate surrogate model (Massengill et al., 1979). To account for original anatomy, authentic material properties and clinical relevance, human metacarpal bones 2 to 5 were obtained from an anatomy department. The donors (three female, two male) had a mean age of 78.3 years, and bone specimens were harvested bilaterally. With prior informed consent of all donors, ethical approval was not required. Next, all specimens were randomly assigned to a group and scanned using a commercially available first-generation Photon Counting CT scanner (Naeotom Alpha, Siemens Healthineers, Forchheim, Germany) to measure the exact length and inner diameter. The specimens were then embedded in cuboid molds using Technovit® 4,071 (Kulzer GmbH, Hanau, Germany). A static universal testing machine (Zwick 2.5 RetroLine, ZwickRoell, Ulm, Germany) and a modified three-point-bending test-setup were used to generate an oblique shaft fracture (Doht et al., 2012). The actuator of the testing machine lowered at a constant speed of 10 mm/min until fracture occurred. Osteosynthesis was performed using partially threaded 4.0 CCS for group 1, fully threaded 4.0 CCS for groups 2 and 4 and a 2.0 TriLock hand plate for groups 3 and 5 (all Medartis GmbH, Basel, Switzerland). The group size was set to 10 after sample size calculation. In the first phase of the study, experimental validation of the fracture model and biomechanical assessment was performed using porcine specimens. Based on the results from these tests, which demonstrated a higher ultimate failure load for fully threaded screws, and taking the limited availability of human specimens into account, only this screw type was selected for subsequent biomechanical testing with human specimens. Correct implant size and positioning were verified by X-ray fluoroscopy after each step, and implants were kept moist throughout preparation and testing.

### 2.2 Biomechanical testing

**FIGURE 1:**
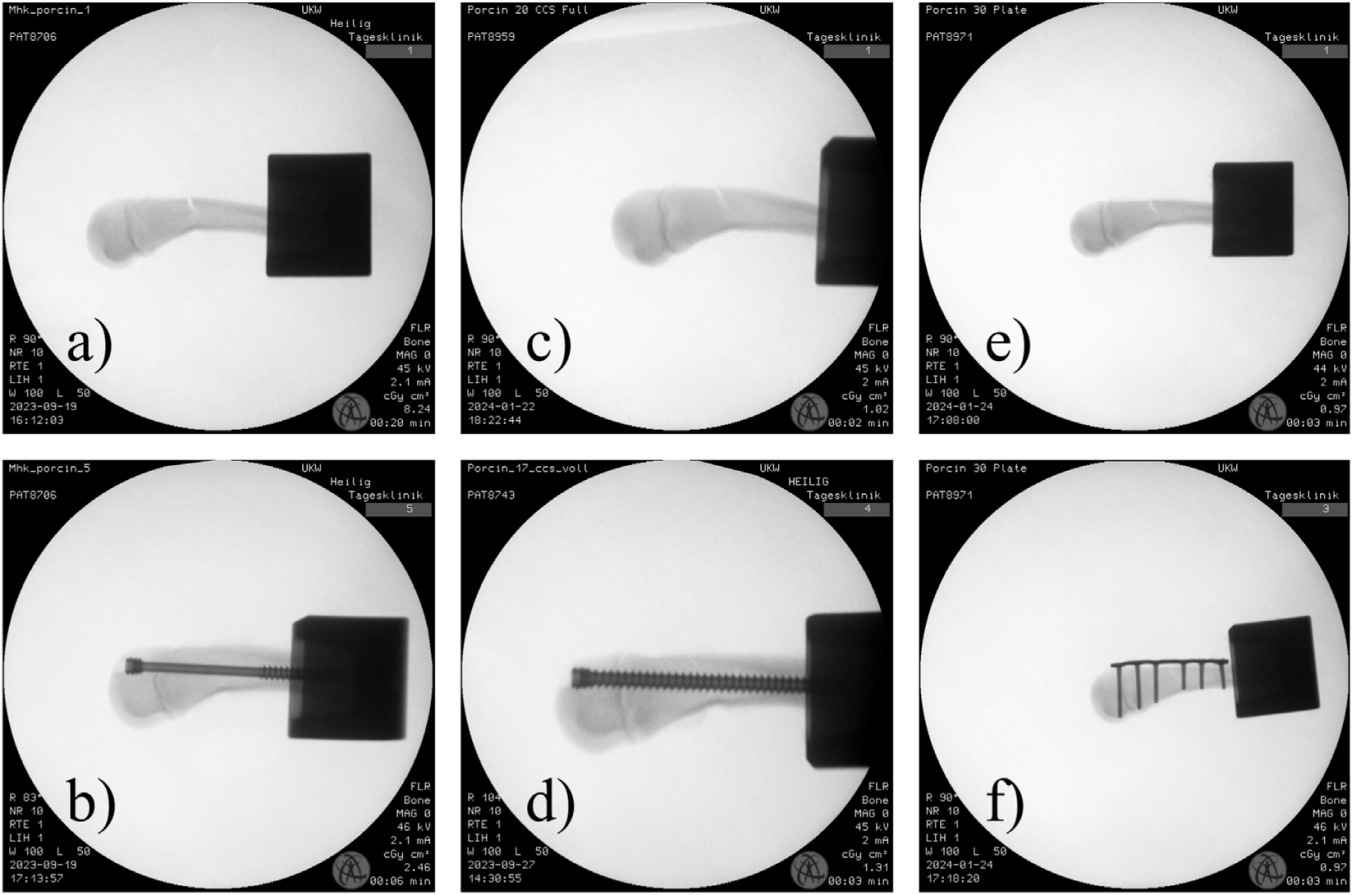
X-rays of porcine specimens of groups 1, 2 and 3 showing the different used implants. **(a,c,e)** showing the generated fractures. **(b-d)** show a specimen treated with an either partially or fully threaded CCS. The entry point was chosen in the dorsal third of the joint surface to minimize cartilage damage. **(f)** shows a specimen which was treated using the 2.0 TriLock hand plate. It was aimed to achieve an almost bicortical length of the screws.

Prior to biomechanical testing, the specimens were rigidly remounted in the modified three-point-bending test. Cyclic loading was applied using the static universal testing machine, with loads ranging from 10 to 80 N for 3,000 cycles at a speed of 100 mm/min corresponding to a frequency of 0.6 Hz. This was chosen to approximate physiologically relevant forces, such as flexor tendon loads of 10–30 N during flexion and up to 120 N during pinch while allowing assessment of construct stability and fatigue resistance without exceeding peak forces (Schuind et al., 1992; Tannen et al., 2017). After completing 3,000 cycles, a load-to-failure test was performed at a speed of 10 mm/min to simulate a worst-case scenario (Figure 1).

### 2.3 Data acquisition and analysis

The static universal testing machine recorded force, displacement, and time at a frequency of 100 Hz. A highly isolated BNC cable connected the machine to the optical system (ARAMIS 3D Professional, Carl Zeiss GOM Metrology GmbH, Braunschweig, Germany), enabling surface strain measurements and documentation of the ongoing testing. Sample size was calculated after initial testing using G*Power 3.1 (Heinrich-Heine-Universität, Düsseldorf, Germany) and revealed a group size of 10 at an effect power of 0.9. Statistical analysis was performed using SPSS V.28 (IBM, NY, United States). Descriptive statistics were first used to calculate mean values and standard deviations for all groups. Then, Shapiro-Wilk and Kolmogorov-Smirnov tests were applied to assess normal distribution. If normal distribution was given, Levene’s test was used to determine whether a standard or Welch-ANOVA should be conducted. For normally distributed data, Tukey’s post-hoc test was applied after ANOVA, while the Games-Howell post-hoc test was used following Welch-ANOVA. If normal distribution was not given, Kruskal-Wallis and Mann-Whitney-U tests were used for analysis.

**FIGURE 2:**
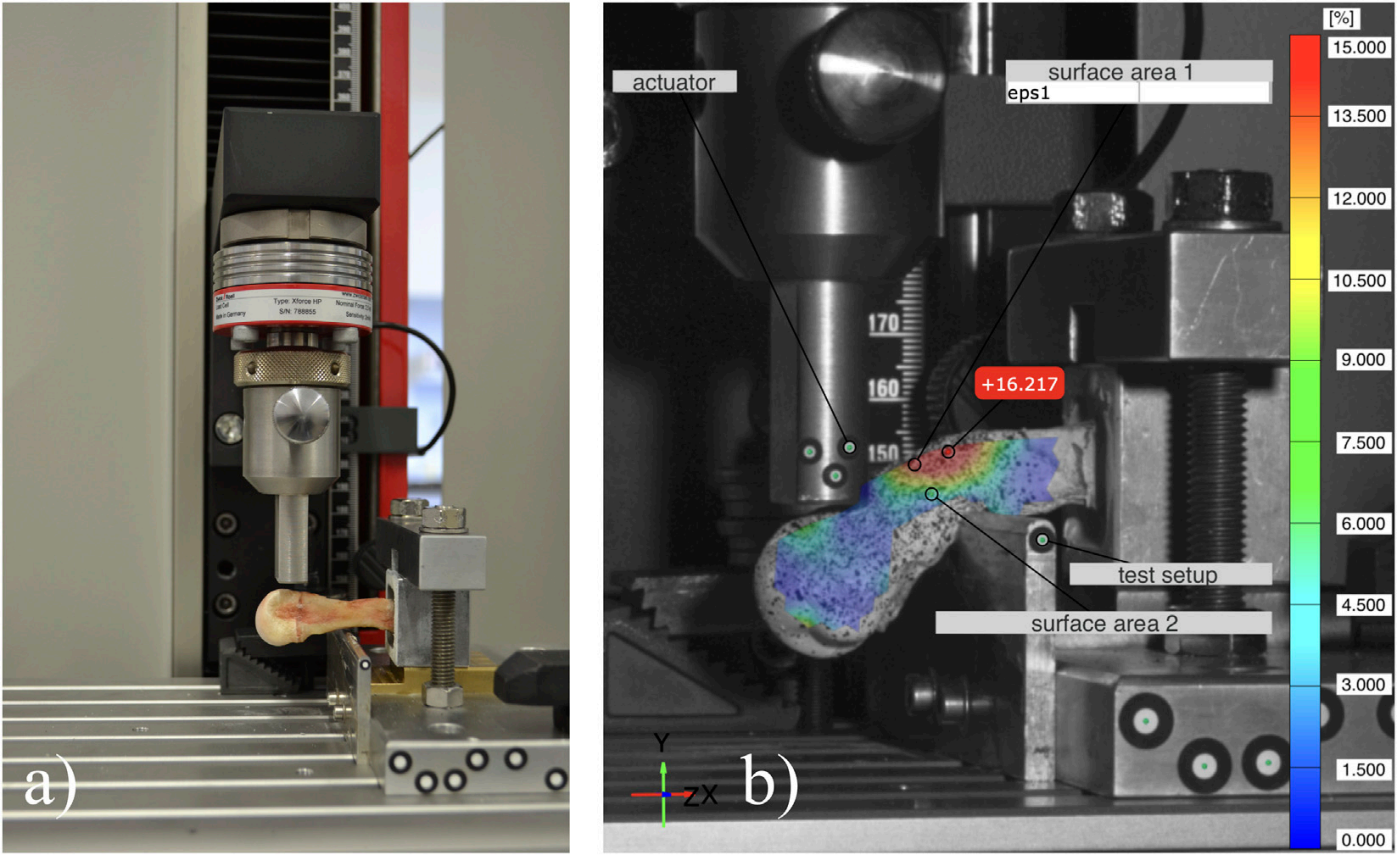
**(a)** The modified 3-point-bending test setup showing a specimen fixated in a cuboid block and positioned beneath the actuator of the Universal Testing Machine. **(b)** The picture shows the optical surface strain measurement at the point of fracture using a stochastic pattern.

The exact significance was calculated for the latter tests since the number of specimens per group was less than 30. A significance level of p < 0.05 was set for all statistical tests. Data visualization was performed using OriginPro® 2021 (OriginLab Corporation, Northampton, MA, United States) (Figure 2).

## 3 Results

### 3.1 Dimensions

Mean values and standard deviations for length and diameter of the used specimens are listed in Table 1. There were no differences found within either the porcine or the human groups.

**TABLE 1:** Overview of the used specimens, their dimensions and the used implants.

Group	Specimen	Implant	Length [mm]	Intramedullary canal diameter [mm]
1	Porcine	4.0 CCS partially threaded	61.1 ± 3.5	6.2 ± 1.4
2	Porcine	4.0 CCS fully threaded	60.1 ± 2.5	6.7 ± 1.2
3	Porcine	2.0 TriLock hand plate	61.3 ± 2.7	6.1 ± 0.7
4	Human	4.0 CCS fully threaded	51.5 ± 6.2	5.1 ± 0.9
5	Human	2.0 TriLock hand plate	53 ± 7	5 ± 0.6

### 3.2 Fracture generation force and displacement

**FIGURE 3:**
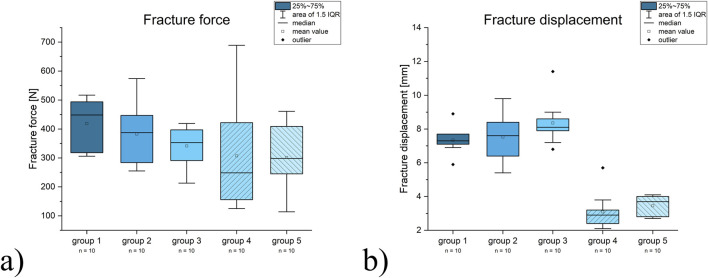
Diagrams for the results for **(a)** fracture force and **(b)** fracture displacement. Statistically significant results are marked with an asterisk.

Fracture generation force was 418.7 ± 86.7 N for group 1, 382.5 ± 105 N for group 2, 341.6 ± 65.4 N for group 3, 307.2 ± 192.7 N for group 4 and 302 ± 119.2 for group 5. The displacement at the moment of fracturing was 7.3 ± 0.8 mm for group 1, 7.5 ± 1.3 mm for group 2, 8.4 ± 1.3 mm for group 3, 3.1 ± 1.1 mm for group 4 and 3.5 ± 0.6 mm for group 5. There were no differences found between groups (Figure 3).

### 3.3 Cyclic displacement and ultimate failure load

The peak-to-peak displacement after cyclic loading was 0.61 ± 0.19 mm for group 1, 0.59 ± 0.22 mm for group 2 and 0.36 ± 0.14 mm for group 3. Human specimens displayed mean values of 1.3 ± 1.12 mm for group 4 and 1.12 ± 1.21 mm for group 5. The displacement for group 3 was significantly lower than for group 1. Notably, one specimen in group 2 failed osteosynthesis and was therefore not available for further testing, and one specimen in group 5 failed in cycle 283 during cyclic loading.

**FIGURE 4:**
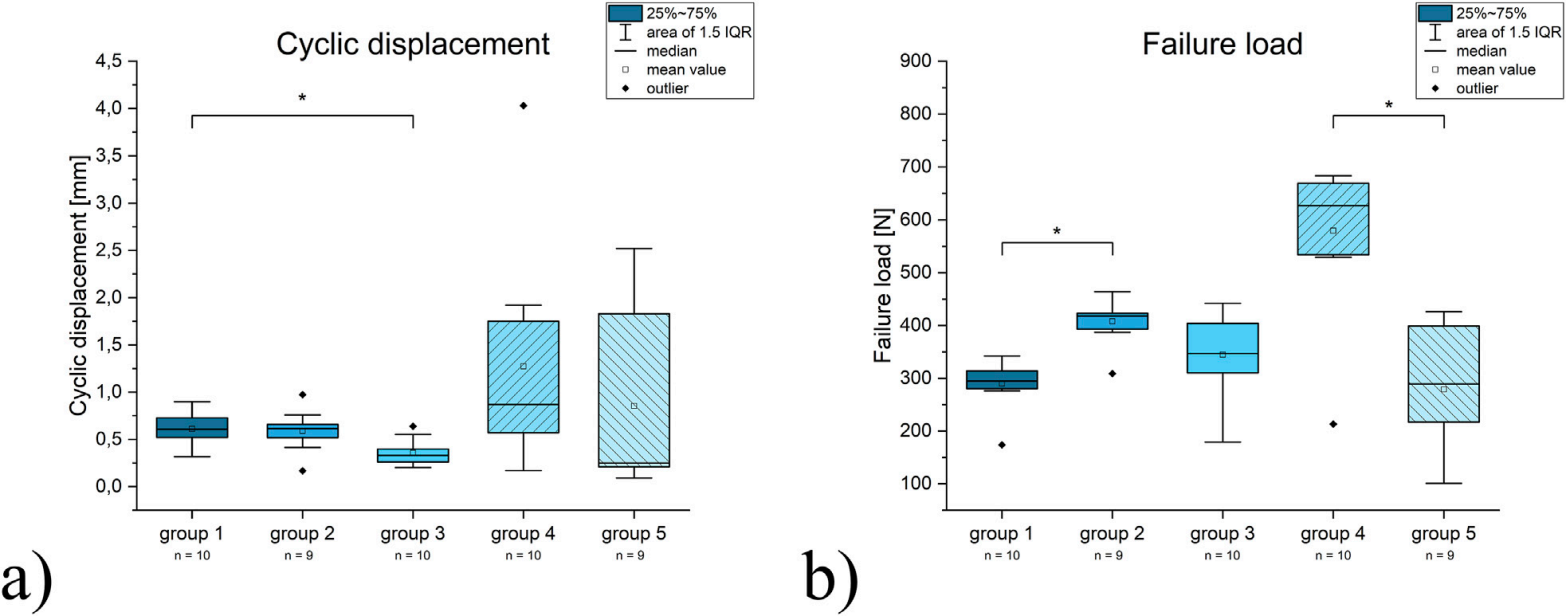
Diagrams for the results for **(a)** cyclic displacement and **(b)** ultimate failure load. Statistically significant results are marked with an asterisk.

Regarding the ultimate failure load, group 1 did show a mean value of 290 ± 46 N, group 2 of 407.7 ± 43.9 N, group 3 of 344.7 ± 80.3 N, group 4 of 580 ± 141.8 N and group 5 of 278.8 ± 120.6 N. The results of group 1 differed significantly to group 2 and the results of group 4 to group 5 (Figure 4).

### 3.4 Displacement at implant failure

**FIGURE 5:**
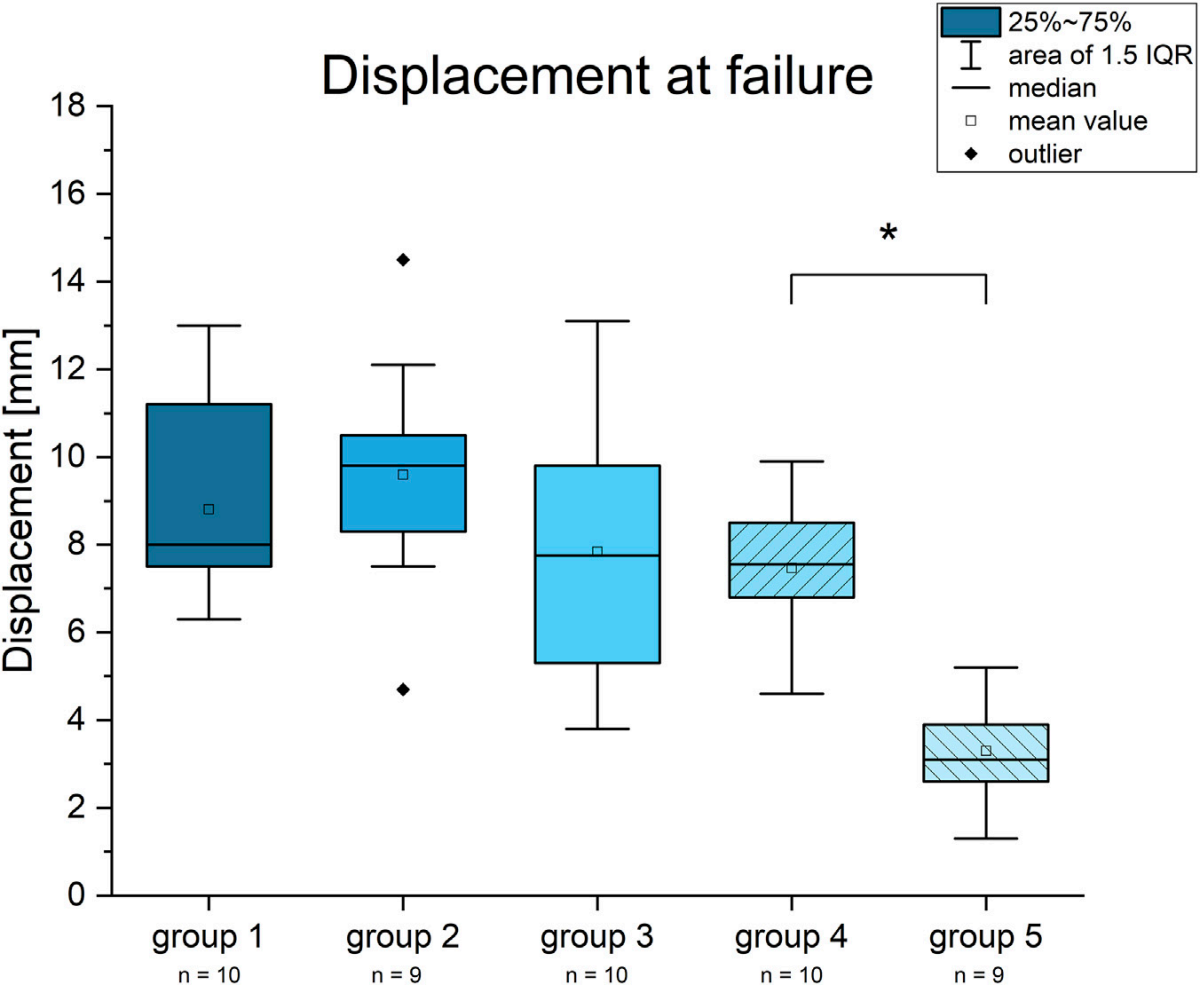
Diagram for the displacement at implant failure. Statistically significant results are marked with an asterisk.

The displacement at the point of implant failure was 8.8 ± 2.3 mm for group 1, 9.6 ± 2.8 mm for group 2, 7.8 ± 3 mm for group 3, 7.5 ± 1.5 for group 4 and 3.3 ± 1.2 for group 5. The results for group 4 were statistically significant to group 5 (Figure 5).

## 4 Discussion

Our study aimed to evaluate the biomechanical stability of a relatively new osteosynthesis technique for metacarpal fractures under cyclic loading conditions, chosen to simulate physiological load as closely as possible in an *ex vivo* setting. While all three techniques successfully achieved osteosynthesis, porcine specimens treated with a plate showed the least displacement after cyclic loading. However, no superiority in terms of a higher ultimate failure load was found for the plate, with the fully threaded CCS performing better in this regard. Relatively, porcine specimens treated with a fully threaded CCS withstood higher failure loads compared to those treated with a partially threaded CCS.

Several factors may explain these findings. First, from a biomechanical perspective, an angle-stable plate with six bicortical screws is a more rigid construct than a single intramedullary screw, whose stability relies primarily on the friction generated between the screw threads and the cortical bone. Second, placing the plate dorsally automatically results in high resistance to downward axial forces. Third, the failure mechanisms observed for plating under ultimate loading were dislocation of the screws out of the bone or thread tearing at the angle stable screw-plate-interface but never plate breakage. Altogether, this indicates that osteosynthesis by plating provides greater initial stiffness but no higher resistance to ultimate failure. When comparing partially and fully threaded CCS, the latter seemed to act more like an intramedullary bolt or strut, with a larger cortical contact area, allowing it to withstand higher ultimate forces. Although cyclic displacement was relatively low for porcine specimens treated with the 2.0 TriLock hand plate, the displacement for those treated with a CCS ranged from 0.32 mm to 0.94 mm. This range represents a sufficiently low amount of movement at the fracture site, which still allows for fracture healing. In direct comparison to human specimens, cyclic displacement was higher with the CCS, possibly due to the human donors being older and having a less healthy cancellous bone stock at the moment of donation compared to young porcine specimens.

The limitations of this study include the use of a modified three-point bending test, which generated primarily bending-dominated loading. *In vivo*, however, physiological loading conditions are known to be multiaxial, involving combined bending, torsion, and axial compression. Therefore, generalizability is limited. Nevertheless, the chosen test setup was considered appropriate, as it successfully reproduced catastrophic clinical failure modes observed under the applied loading conditions. In addition, the model did not account for fracture healing. Second, due to the feasibility of the study, we chose 3,000 cycles as the cyclic loading range, which likely represents only the first few days post-surgery (Galbraith et al., 2022). Third, the test setup required minimal adjustment along the x-axis for some of the human specimens to achieve reproducible fracture generation and to allow for continuous cyclic loading due to length differences between human and porcine specimens. Care was taken to ensure that the relative dimensions of the experimental setup were preserved.

As described by Okoli et al. and Dunleavy et al., a key factor for successful fracture treatment is the selection of the correct screw diameter and length for the metacarpal bone to ensure maximal endosteal purchase (Okoli et al., 2022; Dunleavy et al., 2022). In addition, Allen et al. (Allen et al., 2025) have shown that the screw-to-canal diameter is particularly important for the screw’s stability. This is both supported by our results, as absolute failure loads were higher for fully threaded CCS (groups 2 and 4), and, in relative terms, for the shorter human specimens with a smaller intramedullary canal (group 4). This may be explained by the fact that a fully threaded screw provides a longer segment of thread–bone contact, allowing forces to be distributed over a larger surface area, and that a smaller intramedullary canal enhances load transfer by providing tighter engagement between the intramedullary implant and the surrounding cortical bone. Accordingly, preoperative CT imaging is recommended in clinical practice to determine the optimal screw length and diameter.

In summary, the results of our study indicate that the treatment of metacarpal fractures with cannulated compression screws appears to be non-inferior in comparison to plate fixation from a biomechanical point of view. Accordingly, this technique may provide an alternative to improve fracture management, reduce complication rates, and lower overall healthcare costs.

## 5 Conclusion

The present study contributes to the biomechanical evidence supporting the treatment of metacarpal fractures using cannulated compression screws. CCS, and particularly fully threaded CCS, appear to provide an osteosynthesis that does not fail earlier than a plate in a worst-case scenario. By avoiding the complications associated with plate fixation, this osteosynthesis technique appears to be an increasingly viable alternative.

## Data Availability

The raw data supporting the conclusions of this article will be made available by the authors, without undue reservation.
